# Tumor DNA-dependent protein kinase catalytic subunit expression is associated with hepatitis B surface antigen status and tumor progression in patients with hepatocellular carcinoma

**DOI:** 10.1038/s41598-018-33427-6

**Published:** 2018-10-09

**Authors:** Takayuki Shimizu, Taku Aoki, Shozo Mori, Yukihiro Iso, Masato Kato, Mitsuru Ishizuka, Keiichi Kubota

**Affiliations:** Second Department of Surgery, Dokkyo Medical University, Tochigi, Japan

## Abstract

The DNA-dependent protein kinase catalytic subunit (DNA-PKcs), which plays an important role in the DNA damage response, has been reported to be associated with tumor progression in various carcinomas. However, the clinical significance of DNA-PKcs in hepatocellular carcinoma (HCC) patients remains unclear. In the present study, we determined the tumor expression of DNA-PKcs in 104 resected HCC specimens by immunohistochemistry. The DNA-PKcs expression was scored as follows; 0, negative staining; 1, staining of nuclei at the tumor edge; 2, staining of the nuclei deep within the tumor and/or the tumor cytoplasm. The relationships between tumor expression of DNA-PKcs and the clinical characteristics and patient outcomes were investigated. Among the 104 HCCs, the distribution of staining for DNA-PKcs was as follows: 32 tumors were assigned a score of 0, 27 tumors were assigned a score of 1, and 45 tumors were assigned a score of 2. Statistical analyses revealed that tumor DNA-PKcs expression was significantly associated with the HBs antigen (HBsAg) status, presence/absence of portal vein invasion, size of the largest tumor nodule (<3.0 cm/>3.0, cm), and the serum alpha-fetoprotein level. Significant differences in the overall survival and recurrence-free survival were observed between patients showing (staining score 1 or 2) and not showing (staining score 0) tumor DNA-PKcs expression (*P* = 0.049 and *P* = 0.045, respectively). Our results suggest that tumor expression of DNA-PKcs is associated with tumor progression, HBsAg status and the postoperative outcomes in patients with HCC.

## Introduction

Hepatocellular carcinoma (HCC) is one of the most common cancers encountered worldwide, and the third leading cause of cancer-related death^1,2^. Hepatitis B and hepatitis C are well-known risk factors for the development of HCC^3^. Although antiviral therapies directed against hepatitis B and C have been successfully developed, the risk of development of HCC in patients with these hepatitis has not yet been completely eliminated^4,5^. Since the precise mechanism of development of HCC in patients with hepatitis B or C have not yet been clearly resolved, investigations directed at elucidation of the precise mechanism of development of HCC in patients with hepatitis are important.

DNA double-strand breaks (DSBs) are among the most harmful types of DNA damage to cell survival, as they can arrest the progression of the cell-cycle. DSBs can occur during the DNA replication process, or during exposure to oxidant stress, chemotherapy or radiation^6^. Accumulation of DSBs causes cell death due to genomic instability and chromosomal translocation^6^. There are two cellular pathways available for the repair of DSBs, namely, non-homologous end joining (NHEJ) and homologous recombination (HR)^7^. Although NHEJ is available in all phases of the cell-cycle, as compared to HR which is available only in the S and G2 phases, the accuracy of DNA repair by NHEJ is inferior to that by HR^8^. The DNA protein kinase catalytic subunit (DNA-PKcs) plays an important role in NHEJ. The Ku heterodimer binds broken DNA ends, promoting the recruitment and activation of DNA-PKcs^9^. Activated DNA-PKcs phosphorylates and alters the activities of proteins for NHEJ, including artemis, the X-ray cross complementing protein 4 (XRCC4), the XRCC4-like factor, and DNA ligase IV. As a result of phosphorylation of these proteins, the end of the broken DNA becomes linked to the other end of the DNA^9^. Thus, DNA-PKcs is critical for repair of DSBs.

Tumor DNA-PKcs expression has been shown to be associated with tumor resistance to DNA-damaging therapies, such as radiation^10,11^. Furthermore, DNA-PKcs expression has also been shown to be associated with a poor prognosis in cancer patients, even in the absence of DNA-damaging therapies^12,13^. In addition, DNA-PKcs expression has been reported to be correlated with tumor recurrence and metastasis in prostate cancer^14^. Although the correlation between tumor DNA-PKcs expression and the tumor characteristics is unclear, the aforementioned reports suggest that DNA-PKcs may play a critical role in tumor proliferation.

With regard to hepatocellular carcinoma (HCC), a previous study revealed that DNA-PKcs expression in the tumor is associated with a poor prognosis also in HCC patients^12^. However, the relationships between tumor DNA-PKcs expression and clinical characteristics, such as the presence/absence of underlying hepatitis virus infection, presence/absence of vessel invasion and the disease stage, were not investigated in the previous study. In addition, the relationship between HCC recurrence and tumor DNA-PKcs expression was also not examined. Thus, it still remains unknown how high tumor DNA-PKcs expression leads to a poor prognosis in HCC patients. In the present study, we investigated the relationships between tumor DNA-PKcs expression, as assessed by immunohistochemistry, and the clinical characteristics in patients with HCC.

## Results

### DNA-PKcs expression in the HCCs

**Figure 1:**
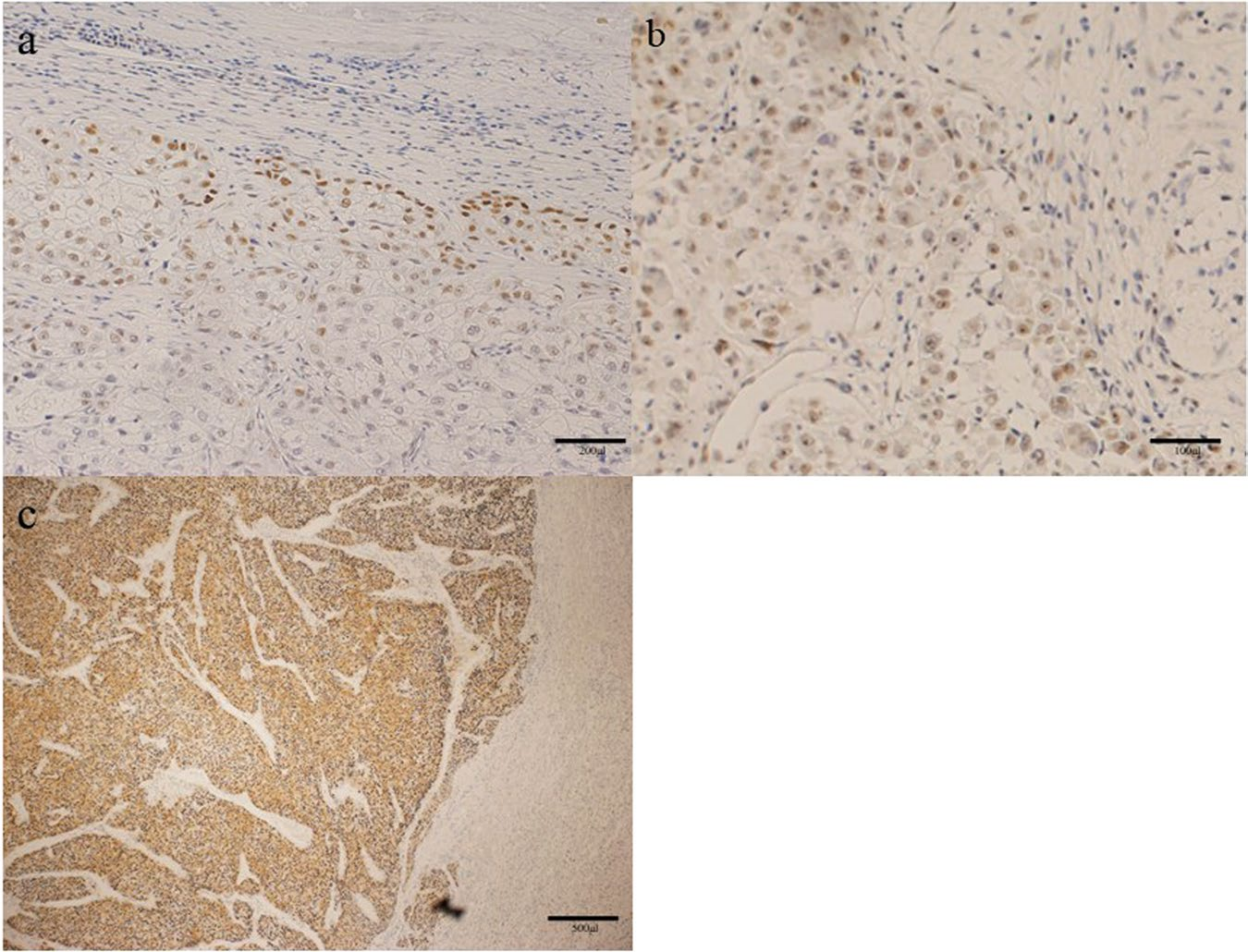
Immunohistochemistry of HCC tumors: The nuclei at the tumor edge were stained in most of the HCC tumors (**a**); staining of the nuclei at the central part of the tumor (**b**); staining of the cytoplasm and the nuclei within the tumor (**c**).

Immunohistochemistry revealed staining of the nuclei at the tumor edge in most of the HCC tumors (72/104, 69.2%) (Fig. 1a), with staining seen in the nuclei in the central part of the tumor in 35 tumors (35/104, 33.6%) (Fig. 1b), and staining of both the cytoplasm and nuclei deep within the tumor seen in 26 tumors (26/104, 25.0%) (Fig. 1c).

### Assessment of DNA-PKcs expression in the HCCs

The immunohistochemical staining for DNA-PKcs in the tumors was scored as follows: score 0, negative staining; score 1, nuclei at the tumor edge; score 2, staining of the nuclei in the central part of the tumor or tumor cytoplasm (Fig. 1). In the present study, 45 of the 104 tumors (43.2%) showed a score of 2, 27 (26.0%) showed a score of 1, and 32 (30.8%) showed a score of 0 (Table 1).

**Table 1:** Frequency distribution of immunohistochemical staining of HCC for DNA-PKcs.

Variable	Number of HCC tumors (n = 104)
DNA-PKcs score	
0	32 (30.8%)
1	27 (26.0%)
2	45 (43.2%)
Total	104

DNA-PKcs; DNA protein kinase catalytic subunit.

### Correlation between tumor DNA-PKcs expression and the clinical and pathological characteristics of HCC patients

Table 2 shows the clinical and pathological characteristics of the HCC patients in each of the DNA-PKcs expression score groups. Chi-square and Kruskal-Wallis tests revealed significant intergroup differences in the HBs Ag status (positive/negative), presence/absence of portal vein invasion, size of the largest tumor nodule (≤3.0 cm/>3.0, cm), and the serum AFP level (ng/mL).

**Table 2:** Relationships between clinical characteristics and DNA-PKcs expression in patients with hepatocellular carcinoma undergoing surgery.

Variable	DNA-PKcs score 0 (n = 32)	DNA-PKcs score 1 (n = 27)	DNA-PKcs score 2 (n = 45)	*P*-value
**Age (years)**				
≤65	9	4	19	
>65	23	23	26	**0**.**047**
**Gender**				
Male	24	10	36	
Female	8	17	9	0.277
**HBs Ag**				
Negative	29	25	33	
Positive	3	2	12	**0**.**045**
**HBV Ab**				
Negative	16	12	22	
Positive	16	15	23	0.904
**HCV Ab**				
Negative	16	18	27	
Positive	16	9	18	0.420
**Child-Pugh classification**				
A	26	19	35	
B	6	8	9	
Not available	0	0	1	0.561
**Liver cirrhosis**				
Absence	19	17	29	
Presence	12	10	15	
Not available	1	0	1	0.915
AFP (ng/mL)	161 ± 611	1312 ± 3907	1626 ± 5089	**0**.**017**
PIVKA-II (mAU/mL)	585 ± 1233	5674 ± 24286	4086 ± 11222	0.675
**Degree of tumor differentiation**				
Well or moderately	30	20	34	
Poorly	2	7	11	0.081
**Size of largest tumor nodule (cm)**				
≤3.0	20	7	19	
>3.0	12	20	26	**0**.**018**
**Tumor Number**				
1	26	18	36	
≥2	6	9	9	0.337
**Portal vein invasion**				
Absence	26	24	25	
Presence	6	3	20	**0**.**004**
**TNM Stage**				
I	13	9	14	
II	13	10	15	
III	6	8	16	0.623

Chi-squared and Kruskal-Wallis test, mean ± S.D.

DNA-PKcs; DNA protein kinase catalytic subunit.

AFP; alpha-fetoprotein, PIVKA-II; protein induced by Vitamin K antagonist II.

HBs Ag; hepatitis virus B antigen, HBV Ab; anti-hepatitis B virus antibody.

HCV Ab; anti-hepatitis C virus antibody, TNM; tumor-node-metastasis.

### Relationship between tumor DNA-PKcs expression and the postoperative outcomes in HCC patients

**Figure 2:**
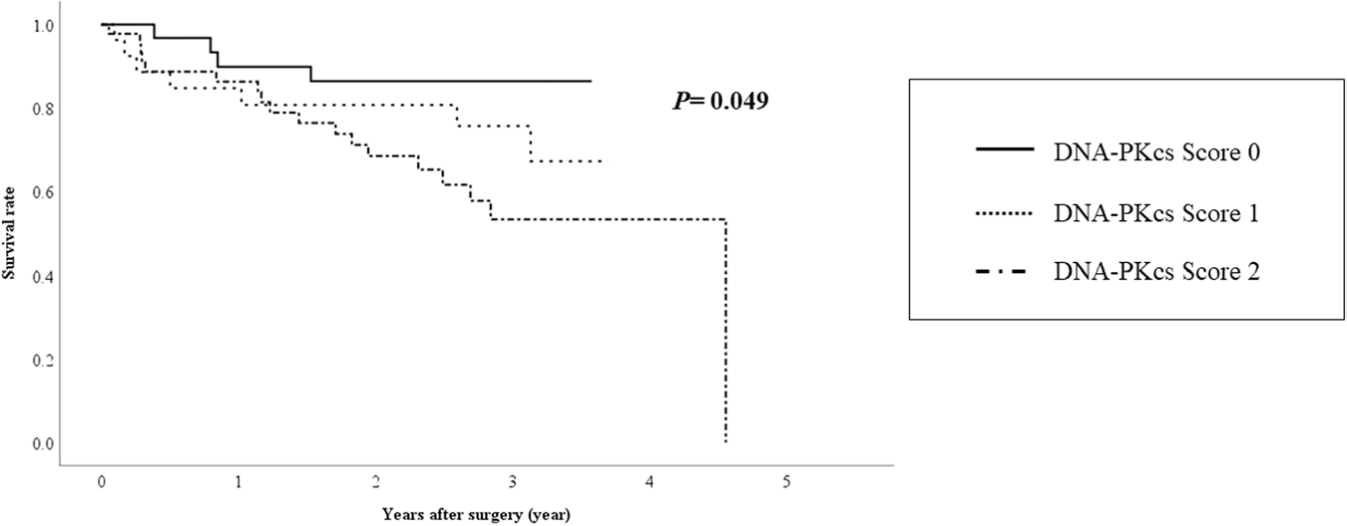
Relationship between DNA-PKcs expression and the overall survival in HCC patients undergoing liver resection.

**Figure 3:**
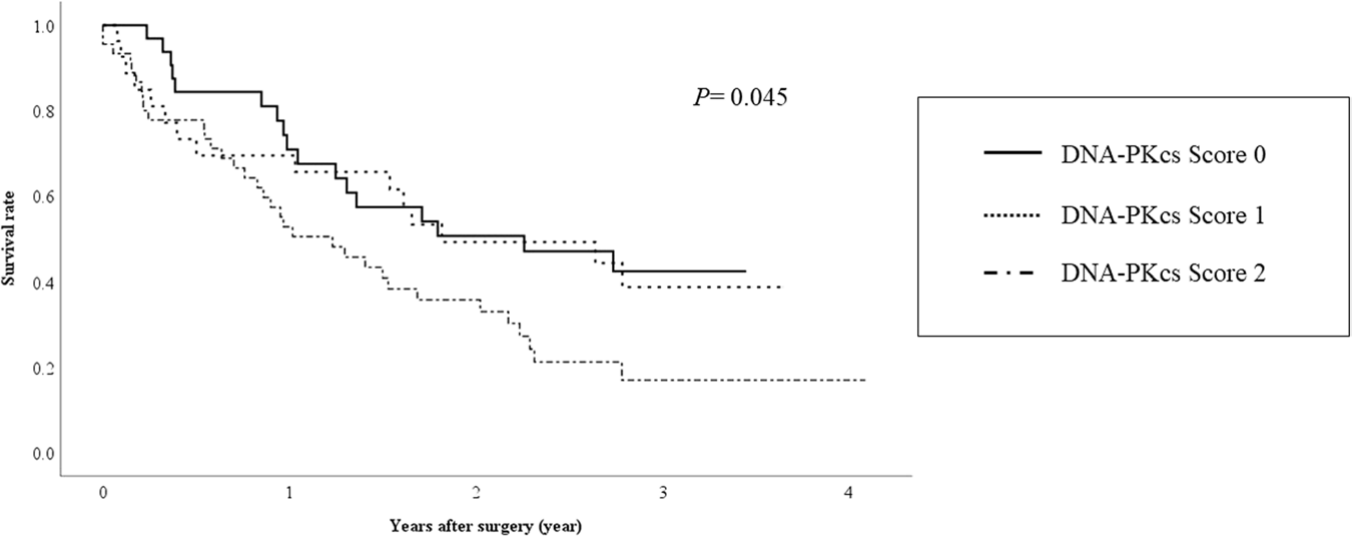
Relationship between the DNA-PKcs expression and the recurrence-free survival in HCC patients undergoing liver resection.

The median and maximum follow-up periods of the survivors were 932 days and 1,663 days, respectively, with a mean survival period of 827 ± 423 days. During the observation period, 29 patients died, including 18 due to cancer and 5 due to liver failure. Kaplan-Meier analysis and comparison by the log-rank test revealed significant differences in the overall survival (OS) (Fig. 2, *P* = 0.049) and recurrence-free survival (RFS) (Fig. 3, *P* = 0.045) among the three DNA-PKcs expression score groups (0, 1, 2).

## Discussion

In the present study, we investigated the tumor DNA-PKcs expression in patients with HCC, including those with underlying hepatitis B and/or hepatitis C. We performed a comprehensive immunohistochemical analysis using a specific antibody against DNA-PKcs. Using this method, we found that tumor DNA-PKcs expression was significantly associated with the HBs Ag status (positive/negative), presence/absence of portal vein invasion, size of the largest tumor nodule (≤3.0 cm/>3.0, cm), serum AFP level (ng/mL), and the postoperative outcomes.

Immunohistochemical analysis showed positive staining of the nuclei at the tumor edge in most HCC tumors (72/104, 69.2%). The aforementioned previous related study made no mention of such a staining pattern in HCC^12^. Because high tumor DNA-PKcs expression was closely associated with elevated serum AFP levels, presence of portal vein invasion and large tumor sizes (>3.0 cm) (Table 2), staining of the nuclei at the tumor edge appears to be associated with tumor progression and growth in HCC tumors. Consistent with the results of the previous study^12^, our results also showed that tumor DNA-PKcs expression in HCC was associated with poor OS and RFS (Figs 1 and 2). HCC patients with high DNA-PKcs expression had poor postoperative outcomes, consistent with our findings that high tumor DNA-PKcs expression was associated with tumor progression and HCC recurrence.

Statistical analysis revealed that tumor DNA-PKcs expression was significantly associated with HBsAg positivity in HCC patients. A recent study has shown that DNA-PKcs is a host protein associated with HBV-RNA^15^. Both HBV-RNA and HBsAg are transcribed by covalently closed circular HBV DNA (cccDNA)^16^. Although nucleotide analogues inhibit viral reverse transcription and viral DNA load^17,18^, episomal viral cccDNA in the nuclei of the infected cells, as well as transcribed production from cccDNA, such as HBV-RNA and HBs Ag, persists even after antiviral therapies^15,16^; thereby cccDNA is a therapeutic obstacle to nucleotide analogue therapies for hepatitis B patients. In fact, HCC risk is determined by the serum levels of HBsAg, and not by those of HBV DNA in HBsAg positive-positive patients^19^. These reports and our own results indicate that cccDNA may contribute to carcinogenesis and tumor progression in HBsAg-positive HCC patients. The relationship between hepatitis virus infection and DNA-PKcs expression in HCCs has never been reported, therefore, this finding is novel and significant.

Several studies have discussed the role of DNA-PKcs in HCC. Suggested mechanisms underlying oncogenesis triggered by DNA-PKcs include hypoxia-inducible factor 1-alpha (HIF1-alpha) activation and oncogene upregulation^12,20–22^. DNA-PKcs expression increased with HIF1-alpha activation, which contributes to hepatocarcinogenesis under hypoxic conditions, in the HepG2 human hepatoma cell line^20,23^. DNA-PKcs also stabilized c-Myc oncoprotein via the Akt/GSK-3beta signaling cascade in the HepG2 cell line^21,24^. In addition, Evert *et al*. have demonstrated that heat shock protein factor-1 induced DNA-PKcs upregulation through the activation of the MAPK/JNK/Activator protein-1 axis. They have shown that activated DNA-PKcs positively correlated with HCC proliferation, genomic instability and microvessel density, and negatively with apoptosis and patients’ survival, and these effects were counteracted by DNA-PKcs silencing^12^. It is unclear how often these phenomena actually occur in human HCC, because these studies were conducted using cell lines. However, pathological assessment of human HCCs revealed that DNA-PKcs expression was directly correlated with genomic instability, the proliferation index, and angiogenesis, and also with suppression of apoptosis^12^. Altogether, these findings suggest that DNA-PKcs could serve as a therapeutic target in HCC.

The major limitation was that the present study was a retrospective study conducted at a single institution. We could not exclude the influence of bias introduced due to the retrospective design of the study. In conclusion, tumor expression of DNA-PKcs in HCC patients was associated with tumor progression, the HBsAg status and postoperative outcomes in HCC patients. However, the mechanisms by which DNA-PKcs triggers carcinogenesis and contributes to tumor progression in cases with HCC remain to be elucidated, and further studies are required.

## Methods

### Study population and follow up of patients

From January 2012 to December 2015, 150 liver resections were performed for HCC at the Second Department of Surgery, Dokkyo Medical University Hospital. Among these, 104 HCC patients were enrolled in this study, because of the availability of good-quality pathological assessment data. The 104 patients included 77 males and 27 females, with a mean age of 68.7 ± 9.0 (mean ± standard deviation [S.D.]) years (range 33–85 years). Seventeen patients were positive for hepatitis B surface antigen (HBsAg), 20 were positive for hepatitis B core antibody (HBcAb) and 26 were positive for anti-hepatitis C virus antibody (HCVAb), 17 were positive for both HBcAb and HCVAb, and 24 were negative for all hepatitis markers.

Routine postoperative surveillance was usually carried out every 3 months in the operated patients. In order to detect any recurrences of the HCC, serum levels of tumor markers, such as alpha-fetoprotein (AFP) and protein induced by vitamin K antagonist II (PIVKA-II), were measured every 3 months after the surgery. In addition, helical dynamic computed tomography (HDCT) was also performed every 3 months, or when the serum tumor marker levels exceeded the normal range. However, when more than 3 years had elapsed after the surgery, the interval at which HDCT was performed was increased from 6 to 12 months; under this circumstance also, HDCT was performed in the event of elevation of the serum tumor markers levels.

### Primary Antibody and Immunohistochemistry

Resected liver specimens were fixed in 10% v/v formalin, cut into blocks, and embedded in paraffin. The blocks were sliced into 4 μm-thick sections and stained with hematoxylin and eosin, or used for immunohistochemical analysis. The DNA-PKcs antibody used for the analysis was a mouse monoclonal antibody (#12311, Cell Signaling Technology, USA). The sections were subjected to dewaxing, heat-induced epitope retrieval with citrate buffer, antibody incubation (dilution 1:30, 45 minutes) and counterstaining on a BOND Max immunostainer using Bond Epitope Retrieval Solution 2 (pH 9.0, 20 minutes) and the Bond Polymer Refine Detection kit (Menarini, Berlin, Germany). In accordance with the suggestion in the manufacturer’s manual, the lymphocytes and bile ducts in the specimen, and spleen were stained as the positive controls.

### Statistical analysis

The correlation between tumor DNA-PKcs expression and various clinical and pathological characteristics in HCC patients were analyzed using the chi-square or Kruskal-Wallis test, as appropriate. The following clinical and pathological characteristics were examined: patient age (≤65/>65, years), gender (male/female), HBsAg status (positive/negative), HBcAb status (positive/negative), HCVAb status (positive/negative), Child-Pugh class (A/B), serum levels of AFP and PIVKA-II, liver cirrhosis (present/absent), degree of tumor differentiation (well/moderately/poorly), size of the largest tumor nodule (≤3.0 cm/>3.0, cm), tumor number (≤1/>1), portal vein invasion (present/absent) and tumor-node-metastasis (TNM) stage in accordance with the Union for International Cancer Control (UICC) classification, 8^th^ edition^25^. Kaplan-Meier analysis and log-rank test were performed to investigate the relationship between tumor DNA-PKcs expression and the postoperative outcomes in HCC patients. All statistical analyses were performed using IBM SPSS statistics version 25.0 software for Windows (IBM Co., New York, NY, USA), and differences with P values of <0.05 were considered as being statistically significant.

### Approval of the Institutional Review Board

This study was conducted with the approval of the Ethical Review Board of the Dokkyo Medical University Hospital (provided ID number: 28110), in compliance with the Ethical Guidelines for Clinical Research published by the Ministry of Health, Labor and Welfare, Japan (http://www.mhlw.go.jp/seisakunitsuite/bunya/hokabunya/kenkyujigyou/i-kenkyu/index.html). A written informed consent was obtained from each patient enrolled in this study.
